# Integrated analysis of mRNA-single nucleotide polymorphism-microRNA interaction network to identify biomarkers associated with prostate cancer

**DOI:** 10.3389/fgene.2022.922712

**Published:** 2022-07-25

**Authors:** Zhiwen Wang, Xi Zhu, Hongyun Zhai, Yanghai Wang, Gangyue Hao

**Affiliations:** Department of Urology, Beijing Friendship Hospital, Capital Medical University, Beijing, China

**Keywords:** prostate cancer, mRNA, SNP, miRNA, interaction network

## Abstract

**Background:** Prostate cancer is one of the most common malignancies among men worldwide currently. However, specific mechanisms of prostate cancer were still not fully understood due to lack of integrated molecular analyses. We performed this study to establish an mRNA-single nucleotide polymorphism (SNP)-microRNA (miRNA) interaction network by comprehensive bioinformatics analysis, and search for novel biomarkers for prostate cancer.

**Materials and methods:** mRNA, miRNA, and SNP data were acquired from Gene Expression Omnibus (GEO) database. Differential expression analysis was performed to identify differentially expressed genes (DEGs) and miRNAs (DEMs). Gene Ontology (GO), Kyoto Encyclopedia of Genes and Genomes (KEGG) pathway analyses, protein-protein interaction (PPI) analysis and expression quantitative trait loci (eQTL) analysis of DEGs were conducted. SNPs related to DEMs (miRSNPs) were downloaded from the open-source website MirSNP and PolymiRTS 3.0. TargetScan and miRDB databases were used for the target mRNA prediction of miRNA. The mRNA-SNP-miRNA interaction network was then constructed and visualized by Cytoscape 3.9.0. Selected key biomarkers were further validated using the Cancer Genome Atlas (TCGA) database. A nomogram model was constructed to predict the risk of prostate cancer.

**Results:** In our study, 266 DEGs and 11 DEMs were identified. KEGG pathway analysis showed that DEGs were strikingly enriched in focal adhesion and PI3K-Akt signaling pathway. A total of 60 mRNA-SNP-miRNAs trios were identified to establish the mRNA-SNP-miRNA interaction network. Seven mRNAs in mRNA-SNP-miRNA network were consistent with the predicted target mRNAs of miRNA. These results were largely validated by the TCGA database analysis. A nomogram was constructed that contained four variables (*ITGB8*, hsa-miR-21, hsa-miR-30b and prostate-specific antigen (PSA) value) for predicting the risk of prostate cancer.

**Conclusion:** Our study established the mRNA-SNP-miRNA interaction network in prostate cancer. The interaction network showed that hsa-miR-21, hsa-miR-30b, and *ITGB8* may be utilized as new biomarkers for prostate cancer.

## Introduction

Prostate cancer (PCa) is the fifth leading cause of cancer death and the second most commonly diagnosed cancer among men worldwide in 2020 (Sung et al., 2021). In addition to significantly impacting the quality of life and health of patients, advanced PCa imposes a substantial economic burden on individuals and societies (Lu et al., 2021). PCa, a highly heterogeneous disease, is tightly regulated by diverse cytokines, gene expression programs and signal pathways throughout the disease course (Wang et al., 2018; Haffner et al., 2021). However, studies on the underlying pathogenesis and molecular mechanisms still remain largely uninvestigated because of lacking integrated molecular analyses. Hence, despite significant advances in treatment, the overall therapeutic benefits still remain unfavorable (Lin et al., 2020; Asif and Teply, 2021). This underscores the importance of early diagnosis of PCa. Prostate-specific antigen (PSA) is a widely used biomarker for the early diagnosis of PCa. However, the value of PSA screening for early detection of PCa remains controversial due to over-diagnosis and over-treatment (Mottet et al., 2021). Previous studies reported that increased PSA may be associated with benign conditions such as infection and inflammation, and some aggressive PCa tissues do not produce PSA, thus resulting in low diagnostic accuracy (McGrath et al., 2015). Therefore, effective biomarkers are urgently needed to improve the accuracy of early diagnosis of PCa.

MicroRNAs (miRNAs) are an important class of single-stranded, non-coding, and endogenous RNAs with 21–25 nucleotides in length, serving as the molecular carriers of highly specific genetic information (Hill et al., 2014). In recent years, miRNA was believed to be pivotal in the pathogenesis of PCa. For instance, the downregulation of miR-146a could suppress the migration and invasion of PCa cells by regulating *ROCK1* levels (Moustafa et al., 2018). The miRNA seed region, located at the 5′ end of the mature miRNA with 2-8 nucleotides could specifically bind to 3′ untranslated region (3′UTR) of target mRNA, called miRNA recognizing element (MRE) sites (Saunders et al., 2007). Thus, any interference in miRNA-MRE interactions may cause dysregulated gene expression and thereby contribute to human diseases. Single nucleotide polymorphisms (SNPs), the most frequent genetic variation, are essential markers for investigating the genetic basis of human diseases. SNPs in miRNA and MRE may disrupt the base complementary pairing to interfere with the combination of mRNA-miRNA, leading to mRNA destabilization and/or translational inhibition (Kang et al., 2016).

Genome-Wide Association Studies (GWAS) have been widely applied to robustly link SNP genotypes with clinical phenotypes, which have identified 269 common SNPs correlated with higher risk of PCa (Conti et al., 2021). Expression quantitative trait loci (eQTL) analysis has been conducted to explore the impact of known disease-associated genetic variation (mostly SNP) on gene expression changes (Fehrmann et al., 2011; Hernandez et al., 2012). However, most of the existing eQTL analyses only study the association between mRNA and SNP, rarely involving miRNA, thus resulting in an incomplete interaction pattern. Therefore, in this study, we aim to construct an mRNA-SNP-miRNA interaction network by bioinformatics analysis and search for novel biomarkers for PCa.

## Materials and methods

### Microarray datasets

The GEO microarray database was searched using different combinations of the following terms: “prostate cancer,” “adjacent normal prostate tissues” and “benign prostatic hyperplasia.” Adjacent normal prostate tissues and benign prostatic hyperplasia were defined as normal prostate. Based on the search strategy above, we obtained two gene expression datasets (GSE54808, GSE69223), one miRNA expression dataset (GSE60117) and one SNP expression dataset (GSE18333). Detailed information for each dataset was presented in Table 1 (see Supplementary Table S1 for further details).

**TABLE 1:** Details of microarray datasets from GEO database.

GSE	Type	Sample size		Chip
		Prostate cancer	Normal prostate	
GSE54808	mRNA	18	12	Affymetrix Human Gene 1.0 ST Array
GSE69223	mRNA	15	15	Affymetrix Human Genome U133 Plus 2.0 Array
GSE60117	miRNA	56	21	Agilent-021827 Human miRNA Microarray
GSE18333	SNP	33	27	Afymetrix Genome-Wide Human SNP 6.0 Array

### Data preprocessing

We downloaded the matrix files of GSE54808 and GSE69223 datasets to acquire gene expression profiles. Probe names in matrix files were converted into gene symbols according to the appropriate platform annotation files. Missing values of the processed gene expression datasets were imputed using the k-Nearest Neighbor (kNN) algorithm by R package impute. The two gene expression datasets were merged by R package dplyr and a batch correction was conducted to remove technical differences using ComBat from the R package sva. The data processing of miRNA expression profile in GSE60117 dataset was similar to those carried out for gene expression profiles, including ID conversion and missing values imputation.

### Differential expression analysis

Differential expression analysis was conducted between normal and PCa tissues by limma package. Genes and miRNAs with q-value < 0.05 and |log2FC|>1 were defined as differentially expressed genes (DEGs) and differentially expressed miRNAs (DEMs), respectively. Subsequently, DEGs and DEMs were visualized by volcano plots and heatmaps using R package pheatmap.

### Enrichment analysis and protein-protein interaction network

Gene Ontology (GO) and Kyoto Encyclopedia of Genes and Genomes (KEGG) pathway analyses were conducted to test the functional enrichment of DEGs using R packages clusterProfiler, DOSE, org.hs.eg.db and topGO. GO terms were identified in three categories: Molecular Function (MF), Biological Process (BP) and Cellular Component (CC). The pathway annotation was obtained from KEGG pathway information. The results were visualized using R packages ggplot2 and tidyverse. Protein-protein interaction (PPI) network for the identified DEGs was established by the Search Tool for the Retrieval of Interacting Genes (STRING) online database. DEGs that the edge counts of every single gene (degree) were more than five were defined as hub genes. To access more reliable results, the minimum interaction score was set at high confidence (0.700).

### mRNA expression quantitative trait loci analysis

EQTL analysis was conducted to analyze the relationship between DEGs and SNP genotypes in GSE18333 using R package MatrixEQTL. cis-eQTL refers to regulatory variation physically locating near the gene itself, and trans-eQTL refers to regulatory variation residing at locations distant from the genes. cis-eQTLs and trans-eQTLs (with *p* < 0.01) were identified and used for subsequent analysis.

### Establishment of mRNA-single nucleotide polymorphism-microRNA interaction network

SNPs in DEMs target sites (miRSNPs) were downloaded from the open-source websites MirSNP (http://bioinfo.bjmu.edu.cn/mirsnp/search/) and PolymiRTS 3.0 (https://compbio.uthsc.edu/miRSNP/). cis-eQTL and trans-eQTL of mRNA were matched with miRSNPs through overlapping SNPs, thus linking mRNAs to miRNAs. We searched for the effect of miRSNPs on the binding sites in this network from the website MirSNP. Then, we searched for the target mRNAs of miRNA in the above mRNA-SNP-miRNA trios on the online tool Targetscan (http://www.targetscan.org/) and miRDB (www.mirdb.org). Finally, the mRNA-SNP-miRNA interaction network was constructed and visualized using Cytoscape 3.9.0.

### The Cancer Genome Atlas dataset analysis

The gene and miRNA expression profiles of the same subjects were obtained from the Cancer Genome Atlas (TCGA) database, including 52 normal prostate and 497 prostate cancer cases. Then, we conducted differential expression analysis by limma package (q-value < 0.05 and |log2FC|>0.5 were used as thresholds) to validate the biomarkers selected from differential expression results and the mRNA-SNP-miRNA interaction network.

### Statistical analysis

All statistical analyses were performed using R software (version 4.1.0, http://www.r-project.org). Student’s t-test was used to determine statistically significant differences between two groups. Boxplots were generated using R package ggplot2 and ggpubr to describe the distribution of expression levels. The receiver operating characteristic (ROC) curve was plotted to assess diagnostic performance of selected biomarkers, and the area under the curve (AUC) was also reported. Univariable logistic regression analyses were used to evaluate the association between PCa and correlated variables, including age, PSA value and selected biomarkers. Then, variables that with *p*-value less than 0.1 in the univariate analysis or with important clinical significance were entered into the multivariate logistic regression analysis. A nomogram was constructed based on the multivariate logistic regression analysis and internal validation of the nomogram was performed using the 1000-bootstrap resample. The performance of the nomogram was evaluated by discrimination and calibration. The AUC was used to determine discriminative ability of the nomogram. The calibration was graphically assessed by a visual calibration plot, which compares the predicted probability with actual probability of PCa. All tests were two-tailed, and *p*-values of less than 0.05 were considered statistically significant.

## Results

### Differential expression analysis

**FIGURE 1:**
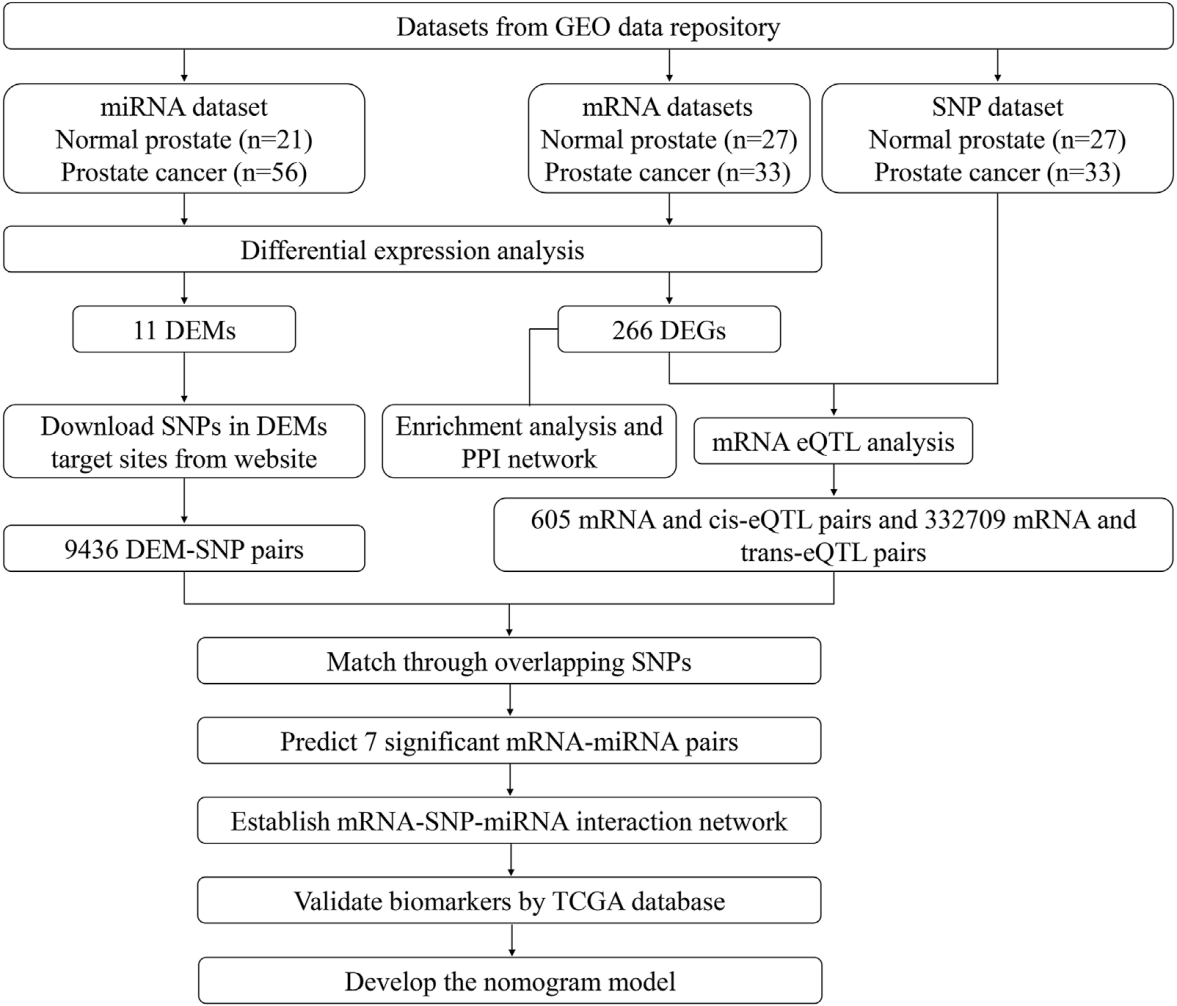
Flow chart of study design.

**FIGURE 2:**
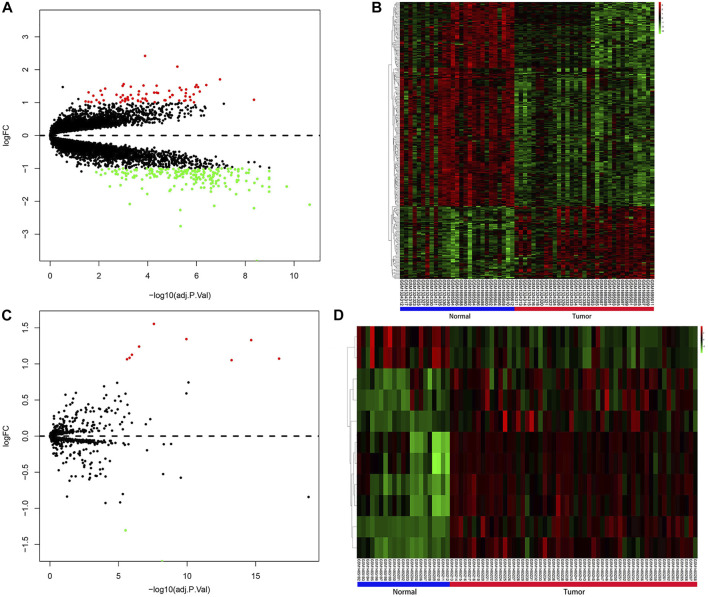
Results of differential expression analysis. **(A)** Volcano plot for DEGs. **(B)** Heatmap for DEGs. **(C)** Volcano plot for DEMs. **(D)** Heatmap for DEMs. Red indicated upregulated DEGs or DEMs, green indicated downregulated DEGs or DEMs, and black indicated genes or miRNAs that were not differentially expressed. In the heatmaps of DEGs and DEMs, the vertical axis represents genes or miRNAs that were clustered by similarity of their transcription profile, and the horizontal axis represents samples. DEGs, differentially expressed genes; DEMs, differentially expressed miRNAs.

The study design and the process of the bioinformatics analysis are shown in Figure 1. In our study, compared with normal prostate tissues, 266 DEGs that FC > 2 or <0.5 and q < 0.05 were screened out, including 70 upregulated and 196 downregulated genes. Sorted by |Log2FC| value, the top 11 DEGs were displayed in Supplementary Table S2. 11 DEMs (|Log2FC |>1 and q < 0.05) were also screened, including 9 upregulated and 2 downregulated miRNAs (Supplementary Table S3). DEGs and DEMs were visualized as heatmaps and volcano plots (Figure 2).

### Enrichment analysis and protein-protein interaction network

**FIGURE 3:**
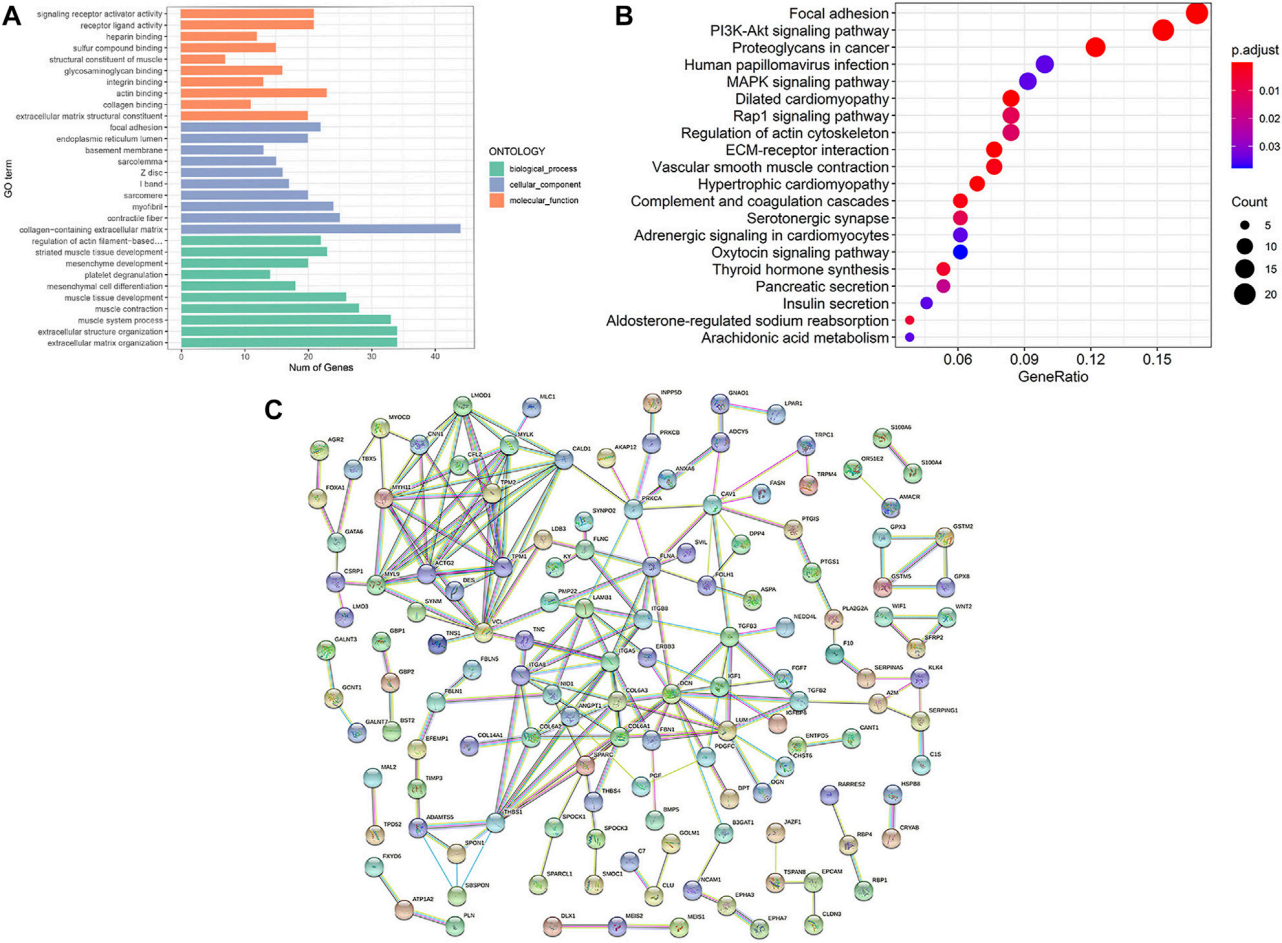
Results of enrichment analysis and PPI network. **(A)** The top 30 enriched significant GO categories of BP, CC, and MF. **(B)** The top 20 enriched KEGG pathways. The magnitude of gene counts is represented by the circle size, and the adjusted *p* value is represented by the legend’s color saturation. **(C)** PPI network of differentially expressed gene. BP, biological process; CC, cellular component; MF, molecular function; PPI, protein-protein interaction.

To study the function of DEGs, we performed GO and KEGG analyses (Figure 3A and Figure 3B). Extracellular matrix organization and extracellular structure organization in BP-associated category, collagen-containing extracellular matrix in CC-associated category and actin binding in MF-associated category were the most significant GO terms enriched with a large number of DEGs. In addition, KEGG analyses showed that DEGs were associated with focal adhesion, PI3K-Akt signaling pathway, proteoglycans in cancer and other molecular pathways. A total of 263 of the 266 DEGs were mapped into the PPI network (Figure 3C). In this network, the parameter degree was used to calculate edge counts of every single gene, and 79 DEGs with the degree>5 were defined as hub genes (Supplementary Table S4).

### mRNA expression quantitative trait loci analysis and mRNA-single nucleotide polymorphism-microRNA interaction network

**FIGURE 4:**
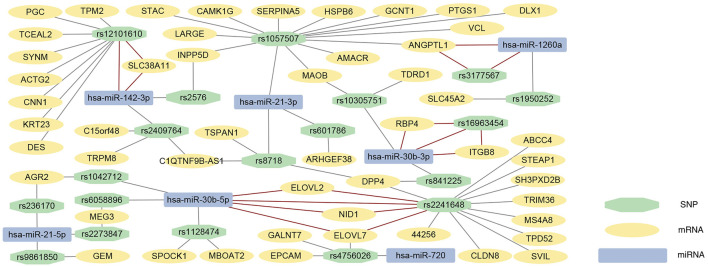
mRNA-SNP-miRNA interaction network. Every box represents one mRNA, SNP or miRNA, detailed in the legend. The 7 mRNA-SNP-miRNA trios with predicted mRNA-miRNA pairs are connected by red solid lines, and other mRNA-SNP-miRNA trios were connected by the grey solid lines. SNP, single nucleotide polymorphism; miRNA, microRNA.

eQTL analysis was conducted to identify the relationship between SNP and mRNA expression, then 605 DEGs and cis-eQTL pairs and 332709 DEGs and trans-eQTL pairs were identified. miRSNPs were acquired from the open-source websites MirSNP and PolymiRTS 3.0 and a total of 9,436 pairs of miRNA-SNPs were screened out. By matching miRNA-miRSNPs pair with cis- and trans-eQTLs of mRNAs through overlapping SNPs, 60 mRNA-SNP-miRNAs trios were identified. The effect and binding energy of binding sites on the miRSNPs in the mRNA-SNP-miRNA trios had been listed in Supplementary Table S5. In addition, we predicted the target mRNAs of DEMs, and 7 target mRNAs in the mRNA-miRNA pairs were found to be in concordance with the mRNAs in our mRNA-SNP-miRNA pairs (Table 2). We ultimately visualized the mRNA-SNP-miRNAs trios by constructing an interaction network using Cytoscape3.9.0 (Figure 4).

**TABLE 2:** Target mRNAs of DEMs in the mRNA-SNP-miRNA network.

DEMs	Target mRNA	Corresponding SNP
hsa-miR-1260a	*ANGPTL1*	rs3177567
hsa-miR-30b-3p	*ITGB8*	rs16963454
hsa-miR-30b-3p	*RBP4*	rs16963454
hsa-miR-30b-5p	*ELOVL7*	rs2241648
hsa-miR-30b-5p	*ELOVL2*	rs2241648
hsa-miR-30b-5p	*NID1*	rs2241648
hsa-miR-142-3p	*SLC38A11*	rs12101610

*ANGPTL1*, angiopoietin like 1; *ITGB8*, integrin subunit beta 8; *RBP4*, retinol binding protein 4; *ELOVL7*, ELOVL fatty acid elongase 7; *ELOVL2*, ELOVL fatty acid elongase 2; *NID1*, nidogen 1; *SLC38A11*, solute carrier family 38 member 11.

### Validation of the selected biomarkers in the Cancer Genome Atlas database

**FIGURE 5:**
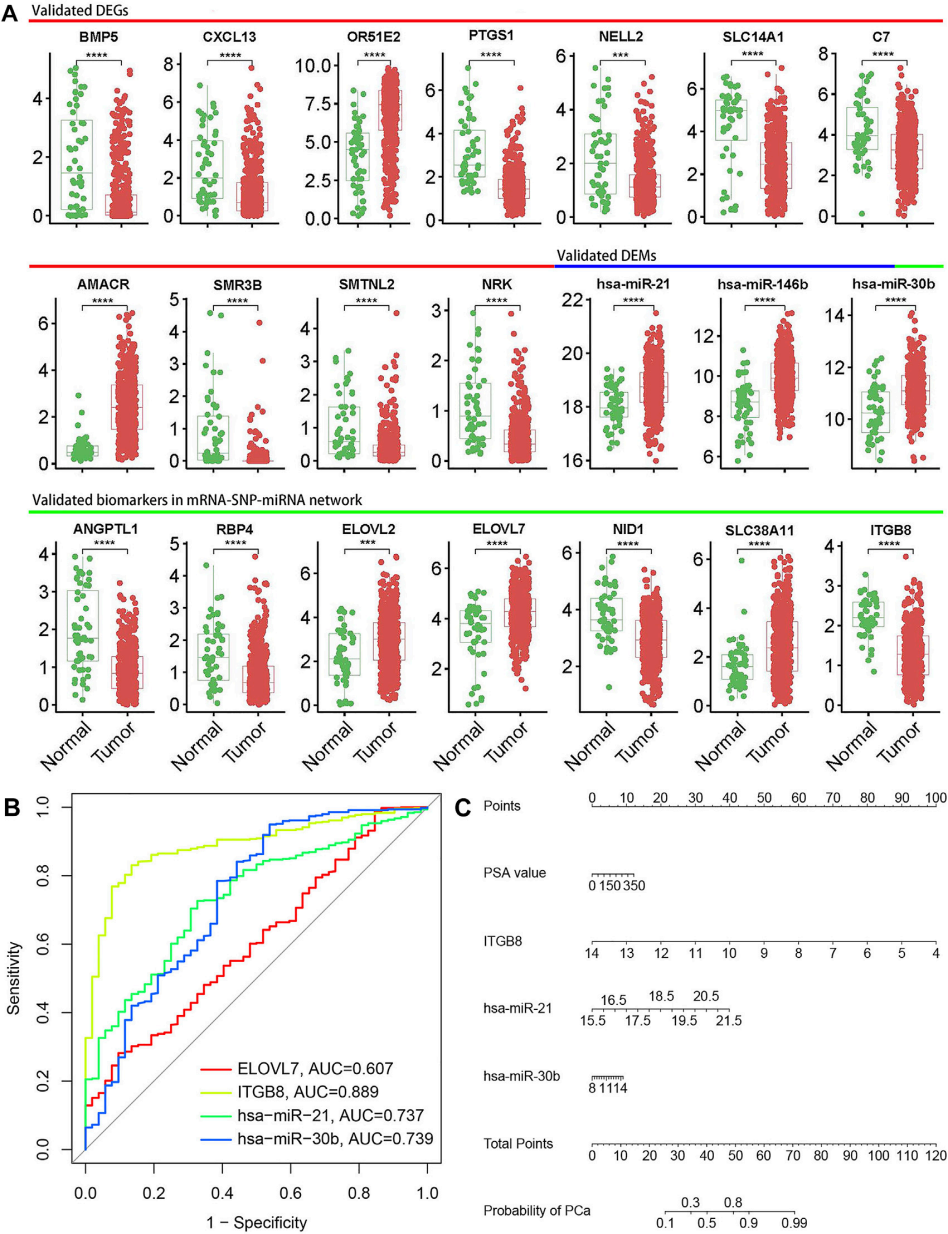
Validation and diagnostic performance of biomarkers. **(A)** Boxplots of representative biomarkers. Significant *p*-values were shown in boxplots; * means *p* value ≤ 0.05, ** means *p* value ≤ 0.01, *** means *p* value ≤ 0.001 and **** means *p* ≤ 0.0001, if not indicated, means *p* value > 0.05. **(B)** Receiver operation characteristic (ROC) analysis to analyze the ability of *ELOVL7*, *ITGB8*, hsa-miR-21, and hsa-miR-30b to distinguish PCa from normal controls. **(C)** Nomogram for predicting the risk of PCa. The points of each variable could be obtained from the point scale axis, then total points could be calculated by summing up each single point. The probability of PCa could be estimated by locating the sum on the total points axis of the nomogram.

The top 11 DEGs and DEMs, and 7 mRNA-miRNA pairs in the interaction network were selected for further validation in the TCGA database. The results showed that all 11 DEGs were confirmed, and three of the top 11 DEMs were validated including hsa-miR-21, hsa-miR-146b-5p and hsa-miR-30b. In addition, all DEGs and one of three DEMs in the 7 mRNA-miRNA pairs were also validated by the TCGA database analysis, including *ANGPTL1*, *RBP4*, *ELOVL2*, *ELOVL7*, *NID1*, *SLC38A11*, *ITGB8*, and hsa-miR-30b. The boxplots for validated biomarkers were presented in Figure 5A. To further explore useful biomarkers for PCa diagnosis, four biomarkers (*ELOVL7*, *ITGB8*, hsa-miR-21, and hsa-miR-30b) were screened out based on the above analysis results and literature survey. The *ITGB8* showed the highest predictive capacity (AUC = 0.889, 95%CI = 0.850–0.927) for PCa, followed by hsa-miR-30b (AUC = 0.739, 95%CI = 0.657–0.820), hsa-miR-21 (AUC = 0.737, 95%CI = 0.672–0.801) and *ELOVL7* (AUC = 0.607, 95%CI = 0.529–0.685) (Figure 5B).

### Nomogram for the probability of prostate cancer

The three biomarkers with AUC>0.7 were selected for model development, and univariable analysis showed that *ITGB8*, hsa-miR-21 and hsa-miR-30b were significantly associated with PCa risk (*p* < 0.05), but age and PSA value were not significant. Considering the significant clinical diagnostic implications of PSA value, *ITGB8*, hsa-miR-21, hsa-miR-30b and PSA value were finally entered into the multivariable logistic regression analysis. Based on the regression analysis, a nomogram was constructed that contained four variables for predicting the risk of PCa (Figure 5C). The nomogram yielded an AUC of 0.919 (95% CI: 0.889–0.949), indicating a good discrimination ability for PCa (Supplementary Figure S1A). The calibration curve showed good agreement among the prediction by the nomogram and actual observations (Supplementary Figure S1B).

## Discussion

Advanced PCa is a lethal illness with a dismal prognosis. Early diagnosis and treatment for PCa may be particularly significant. Although PSA was widely used to screen for PCa, there were several limitations in PSA testing as population screening program. Low sensitivity of PSA testing has limited its application for PCa screening (Sanda et al., 2017). Previous research has reported that 15% of men with PSA of less than 4.0 ng/ml would be diagnosed with PCa, of which 15% would be diagnosed with high-grade PCa (Thompson et al., 2004; Lucia et al., 2008). PSA testing also has specificity limitations. Currently, there were only less than half of men whose PSA level was higher than 4.0 ng/ml were diagnosed with PCa by needle biopsy (Schröder et al., 2009; Tomlins et al., 2011). Understanding the molecular mechanisms of PCa development may contribute to identifying effective biomarkers for this disease. However, the specific regulatory mechanism of miRNA and SNP still remains unclear due to the lack of integrated molecular analyses. Constructing an mRNA-SNP-miRNA interaction network contributes to the comprehensive analysis of the underlying regulatory mechanisms between genetic variants and diseases. Thus, we combined the profiles of mRNA, SNP, and miRNA from GEO microarray datasets and built an interaction network and nomogram model synthetically. Our study has identified several novel biomarkers in the mRNA-SNP-miRNA trios, revealing that SNPs in miRNA and MRE may interfere with the combination of mRNA-miRNA, which is beneficial to explore the novel biomarkers for PCa.

In our present study, 266 DEGs were identified, including 70 upregulated and 196 downregulated genes. *C7* was one of the top-ranked downregulated DEGs in this study based on the |log2FC| values. Interestingly, previous studies have reported similar findings among PCa patients, and demonstrated that *C7* was closely associated with immune infiltration level, overall survival and disease-free survival of the patients, suggesting that *C7* had the potential for use as a prognostic prediction and immunoregulatory target in PCa (Chen, et al., 2020). Moreover, *BMP5* has been found to closely relate to the progression of PCa. Huang et al. (2012) analyzed the genotype of 601 patients with PCa and showed that PCa cell lines displayed high expression of *BMP5*, and rs3734444 in *BMP5* was significantly correlated with PCa-specific mortality (Huang et al., 2012). Another study found that inhibition of *BMP5* can be effective in delaying the PCa progression of Pten-deficient mice by impairing self-renewal capacity of stem/progenitor cells (Tremblay et al., 2020). However, our research showed that *BMP5* was differentially downregulated in PCa patients, which was inconsistent with prior studies. Differences in the experimental approaches, species and specimen types may explain the discordant results, and further experiments are required to validate the findings of this research. Notably, the activation of *OR51E2*, another significantly overexpressed DEG in our study, was able to enhance the proliferation of androgen-independent PCa cells, thus driving transformation to neuroendocrine cancer (Abaffy et al., 2018). Our study provided converging evidence that the upregulated *OR51E2* might be crucial in the pathogenesis of PCa.

MiRNAs have been recognized as a group of non-coding RNA which are intimately involved in posttranscriptional regulation. In our study, 11 DEMs including nine upregulated and two downregulated miRNAs were identified. Consistent with our study, previous research has reported that several upregulated DEMs were implicated in the progression of PCa. Prior findings showed that hsa-miR-21 could regulate the growth of androgen-independent PCa, and only the overexpression of hsa-miR-21 was enough to enable androgen-dependent cancer cells to overcome castration and progress to androgen-independent cancer (Ribas et al., 2009; Fabris et al., 2016; Wang et al., 2020). Therefore, the upregulation of hsa-miR-21 might be associated with ADT-resistance and castration-resistant prostate cancer (CRPC) progression. Since liquid biopsy became more widely applied in PCa, hsa-miR-30b-3p was found high-expressed in the extracellular vesicles of patients’ urine, suggesting that hsa-miR-30b-3p has the potential as a biomarker for PCa (Wang et al., 2020; Matsuzaki et al., 2021). In addition, *circ_CCNB2* knockdown can upregulate hsa-miR-30b-5p to modulate *KIF18A* expression and enhance the radiosensitizing effect for PCa patients (Cai et al., 2020). These DEMs might offer vital insight into underlying regulatory mechanisms of PCa.

KEGG pathway analysis of DEGs revealed significant enrichment for focal adhesion and PI3K-Akt signaling pathway. Focal adhesion kinase (FAK) was an important molecular for focal adhesion signaling pathway and a non-receptor tyrosine kinase that mediates integrin-based signaling. Previous studies suggested that FAK participated in the proliferation, adhesion, migration and survival of PCa cells, suggesting focal adhesion may play a vital role in PCa pathogenesis (Figel and Gelman, 2011). 22 DEGs were associated with focal adhesion signaling pathway, including one upregulated gene (*THBS4*) and 21 downregulated genes (*PRKCA*, *ITGB8*, *MYLK*, *FLNA*, *VCL*, *CCND2*, *MYL9*, *THBS1*, *ITGA5*, *COL6A3*, *LAMB1*, *TNC*, *CAV1*, *FLNC*, *PRKCB*, *COL6A2*, *IGF1*, *PGF*, *COL6A1*, *ITGA8*, *PDGFC*). Of these, the key gene *IGF1* has been reported to drive the fine-tuning network between the integrin-FAK signaling and the Akt-mTOR pathway, thus promoting the growth and invasion of PCa cells (Siech et al., 2022). Furthermore, Shorning et al. (2020) found that PI3k-Akt signaling pathway could interact with other oncogenic signaling cascades, like androgen receptor, thereby stimulating the growth and developing drug resistance of PCa cells (Shorning et al., 2020). *ITGA5* is the key gene in PI3k-Akt signaling pathway. The combination treatment of the pharmaceutical inhibition of PI3K signaling pathway and *ITGA5* knockdown has been reported to promote apoptosis in PTEN mutant PCa cells, possibly due to the joint signal transduction to BCL-X_L_ (Ren et al., 2016). Taken together, it is reasonable to speculate that the focal adhesion and PI3K-Akt signaling pathway were probably relevant to the mechanism of PCa, and *IGF1* and *ITGA5* could be potential markers for PCa.

The mRNA-SNP-miRNA interaction network was comprised of 19 SNPs, 7 miRNAs, and 51 mRNAs. The results were verified by online websites Targetscan and miRDB, and ultimately 7 mRNA-SNP-miRNA trios were found to be related with each other. Among the 7 trios, we found several DEGs and DEMs have important implications for understanding the underlying mechanism of PCa, including hsa-miR-30b, hsa-miR-142-3p, *ITGB8*, and *ELOVL7*. miRNA hsa-miR-30b-5p was found to be related to 17 DEGs through 4 overlapped SNPs, and hsa-miR-30b-3p was correlated with 5 DEGs through 3 SNPs. These findings indicate hsa-miR-30b might contribute importantly to the pathogenesis of PCa. As mentioned above, overexpression of hsa-miR-30b-3p was found in the extracellular vesicles in patients’ urine, and the upregulating hsa-miR-30b-5p can exert a radiosensitizing effect on tumor cells for PCa patients, providing supporting evidence for our study. In addition, hsa-miR-142-3p related to 13 DEGs through 3 SNPs. Hsa-miR-142-3p can bind to the 3′UTR of *FOXO1* to decrease *FOXO1* expression, causing the proliferation of PCa cells and suppression of apoptosis (Tan et al., 2020). However, hsa-miR-142-3p was not found to be differentially expressed in the samples from the TCGA database, and further validation is required to elucidate its biological functions.

In the interaction network, *ITGB8* was associated with hsa-miR-30b-3p through rs16963454. Previous findings have reported that *ITGB8* can be upregulated by miR-93 to promote the proliferation and invasion of PCa cells, confirming the instrumental role of *ITGB8* in PCa carcinogenesis (Liu et al., 2018). However, our results found that *ITGB8* was obviously downregulated in PCa. This is possibly because different miRNA binding activates different pathways, thus follow-up experimental studies are needed to further elucidate its specific mechanism. Moreover, *ELOVL7*, a validated overexpressed DEG in our interaction network, was associated with hsa-miR-30b-5p through rs2241648, which might make a critical contribution to the development of PCa. Specifically, *ELOVL7* may be involved in the growth and survival of PCa cells through the metabolism of saturated very-long-chain fatty acids (SVLFAs) and their derivatives in previous study, which may become a druggable molecular target for novel treatment or prevention strategies (Tamura et al., 2009). Nevertheless, since the AUC of ELOVL7 was only 0.607, we could not determine its diagnostic performance, and further validation is required. Based on the above results, we constructed a nomogram model to predict the risk of PCa pathogenesis. However, we did not find relevant literature directly relating to the regulatory mechanism of the SNPs identified in our study, thus further validation by experimental investigations is needed.

In this study, we constructed an mRNA-SNP-miRNA interaction network and a nomogram model for PCa. However, several limitations still exist in this study. First, Gleason score and neoadjuvant treatment, which may affect gene and miRNA expression in PCa, were not available in our datasets, thus we could not evaluate their association with our results. Second, due to differences in sample and microarray types between the two gene expression datasets, the batch effect cannot be completely removed and may affect the robustness of our results. Third, the GEO datasets of mRNA, SNP and miRNA in this study were not obtained from the same population, which may be subject to bias in the results. To eliminate the bias as much as possible, we performed batch correction and analyzed the gene and miRNA expression profiles of the same subjects from TCGA database to validate our results. In the future, the mRNA, miRNA and SNP datasets from the same samples are still required to offer more compelling outcomes, and our results need further experiments to validate.

## Conclusion

Our study established mRNA-SNP-miRNA interaction network in PCa. In the interaction network, we found hsa-miR-21, hsa-miR-30b and *ITGB8* played vital regulatory roles in the pathogenesis of PCa. Further experiments are needed to verify the specific mechanism of these screened-out biomarkers in PCa.

## Data Availability

The original contributions presented in the study are included in the article/Supplementary Material, further inquiries can be directed to the corresponding author.
