# Supplementary material for: Integrated analysis of mRNA-single nucleotide polymorphism-microRNA interaction network to identify biomarkers associated with prostate cancer
Source: Front Genet. 2022 Jul 25;13:922712. doi: 10.3389/fgene.2022.922712 (PMC9358224; doi:10.3389/fgene.2022.922712)
Supplement: Supplementary file 1 [file DataSheet1.docx]

**Table S1**: Sample IDs

| **GSE** | **Classification** | **GSM** |
| --- | --- | --- |
| **GSE54808** | **Normal prostate** | **GSM1324312 GSM1324317 GSM1324319**  **GSM1324323 GSM1324325 GSM1324327**  **GSM1324328 GSM1324330 GSM1324331**  **GSM1324335 GSM1324336 GSM1324340** |
|  | **Prostate cancer** | **GSM1324311 GSM1324313 GSM1324314**  **GSM1324315 GSM1324316 GSM1324318**  **GSM1324320 GSM1324321 GSM1324322**  **GSM1324324 GSM1324326 GSM1324329**  **GSM1324332 GSM1324333 GSM1324334**  **GSM1324337 GSM1324338 GSM1324339** |
| **GSE69223** | **Normal prostate** | **GSM1695584 GSM1695586 GSM1695588**  **GSM1695590 GSM1695592 GSM1695594**  **GSM1695596 GSM1695598 GSM1695600**  **GSM1695602 GSM1695604 GSM1695606**  **GSM1695608 GSM1695610 GSM1695612** |
|  | **Prostate cancer** | **GSM1695583 GSM1695585 GSM1695587**  **GSM1695589 GSM1695591 GSM1695593**  **GSM1695595 GSM1695597 GSM1695599**  **GSM1695601 GSM1695603 GSM1695605**  **GSM1695607 GSM1695609 GSM1695611** |
| **GSE60117** | **Normal prostate** | **GSM1465192 GSM1465193 GSM1465194**  **GSM1465195 GSM1465196 GSM1465197**  **GSM1465198 GSM1465199 GSM1465200**  **GSM1465201 GSM1465202 GSM1465203**  **GSM1465204 GSM1465205 GSM1465206**  **GSM1465207 GSM1465208 GSM1465209**  **GSM1465210 GSM1465211 GSM1465212** |
|  | **Prostate cancer** | **GSM1465213 GSM1465214 GSM1465215**  **GSM1465216 GSM1465217 GSM1465218**  **GSM1465219 GSM1465220 GSM1465221**  **GSM1465222 GSM1465223 GSM1465224**  **GSM1465225 GSM1465226 GSM1465227**  **GSM1465228 GSM1465229 GSM1465230**  **GSM1465231 GSM1465232 GSM1465233**  **GSM1465234 GSM1465235 GSM1465236**  **GSM1465237 GSM1465238 GSM1465239**  **GSM1465240 GSM1465241 GSM1465242**  **GSM1465243 GSM1465244 GSM1465245**  **GSM1465246 GSM1465247 GSM1465248**  **GSM1465249 GSM1465250 GSM1465251**  **GSM1465252 GSM1465253 GSM1465254**  **GSM1465255 GSM1465256 GSM1465257**  **GSM1465258 GSM1465259 GSM1465260**  **GSM1465261 GSM1465262 GSM1465263**  **GSM1465264 GSM1465265 GSM1465266**  **GSM1465267 GSM1465268** |
| **GSE18333** | **Normal prostate** | **GSM457682 GSM457684 GSM457686**  **GSM457688 GSM457690 GSM457693**  **GSM457695 GSM457697 GSM457699**  **GSM457702 GSM457704 GSM457706**  **GSM457708 GSM457710 GSM457712**  **GSM457714 GSM457716 GSM457719**  **GSM457721 GSM457723 GSM457725**  **GSM457727 GSM457729 GSM457731**  **GSM457733 GSM457737 GSM457739** |
|  | **Prostate cancer** | **GSM457681 GSM457683 GSM457685**  **GSM457687 GSM457689 GSM457691**  **GSM457692 GSM457694 GSM457696**  **GSM457698 GSM457700 GSM457701**  **GSM457703 GSM457705 GSM457707**  **GSM457709 GSM457711 GSM457713**  **GSM457715 GSM457717 GSM457718**  **GSM457720 GSM457722 GSM457724**  **GSM457726 GSM457728 GSM457730**  **GSM457732 GSM457734 GSM457735**  **GSM457736 GSM457738 GSM457740** |

**Table S2.** Top 11 mRNA in absolute value of log2(fold change)

| mRNA | *q*-value | Log2FC | regulation |
| --- | --- | --- | --- |
| *BMP5* | <0.05 | -3.82 | downregulation |
| *CXCL13* | <0.05 | -2.76 | downregulation |
| *OR51E2* | <0.05 | 2.42 | upregulation |
| *PTGS1* | <0.05 | -2.27 | downregulation |
| *NELL2* | <0.05 | -2.21 | downregulation |
| *SLC14A1* | <0.05 | -2.14 | downregulation |
| *C7* | <0.05 | -2.10 | downregulation |
| *AMACR* | <0.05 | 2.09 | upregulation |
| *SMR3B* | <0.05 | -2.08 | downregulation |
| *NRK* | <0.05 | -1.78 | downregulation |
| *SMTNL2* | <0.05 | -1.78 | downregulation |

**Table S3.** miRNA in absolute value of log2(fold change)

| miRNA | *q*-value | Log2FC | regulation |
| --- | --- | --- | --- |
| hsa-miR-1268 | <0.05 | -1.73 | downregulation |
| hsa-miR-1274b | <0.05 | 1.55 | upregulation |
| hsa-miR-1274a | <0.05 | 1.34 | upregulation |
| hsa-miR-1260 | <0.05 | 1.33 | upregulation |
| hsa-miR-1207-5p | <0.05 | -1.30 | downregulation |
| hsa-miR-1308 | <0.05 | 1.24 | upregulation |
| hsa-miR-142-3p | <0.05 | 1.13 | upregulation |
| hsa-miR-720 | <0.05 | 1.08 | upregulation |
| hsa-miR-21* | <0.05 | 1.07 | upregulation |
| hsa-miR-146b-5p | <0.05 | 1.06 | upregulation |
| hsa-miR-30b | <0.05 | 1.05 | upregulation |

**Table S4.** Top 30 hub genes in PPI network

| Hub genes | degree | Hub genes | degree |
| --- | --- | --- | --- |
| *DCN* | 29 | *MYLK* | 20 |
| *IGF1* | 26 | *TIMP3* | 20 |
| *FLNA* | 25 | *TGFB3* | 19 |
| *THBS1* | 25 | *ITGA5* | 18 |
| *CAV1* | 24 | *TPM1* | 18 |
| *FBN1* | 24 | *CSRP1* | 17 |
| *VCL* | 24 | *FBLN1* | 17 |
| *LUM* | 23 | *TGFB2* | 17 |
| *MYH11* | 23 | *TPM2* | 17 |
| *COL6A1* | 22 | *ITGB8* | 16 |
| *FLNC* | 22 | *ACTG2* | 15 |
| *SPARC* | 22 | *PRKCA* | 15 |
| *COL6A2* | 21 | *A2M* | 14 |
| *COL6A3* | 20 | *EPCAM* | 14 |

**Table S5:** relationship between SNPs and DEMs in the mRNA-SNP-miRNA trios

| miRNA_ID | snpid | mirSVR | Effect | Allele | Score | Energy | Conservation |
| --- | --- | --- | --- | --- | --- | --- | --- |
| hsa-miR-21-3p | rs1057507 | -0.64 | decrease | A | 152 | -21.1 | 0.001 |
| hsa-miR-21-5p | rs236170 | -0.941 | enhance | C | 144 | -11.44 | 0.001 |
|  |  |  |  | T | 151 | -13.17 | 0.001 |
| hsa-miR-21-5p | rs2273847 | -1.054 | break | A | 151 | -9.66 | 0.033 |
| hsa-miR-21-3p | rs601786 | -0.57 | decrease | T | 153 | -18.11 | 0.669 |
|  |  |  |  | C | 147 | -18.11 | 0.669 |
| hsa-miR-21-5p | rs9861850 | -0.527 | decrease | G | 146 | -9.41 | 0 |
|  |  |  |  | A | 143 | -9.47 | 0 |
| hsa-miR-21-3p | rs8718 | - | create | G | 148 | -14.29 | 0.001 |
| hsa-miR-1260a | rs1950252 | - | create | G | 159 | -25.15 | 0.011 |
| hsa-miR-1260a | rs3177567 | - | create | G | 151 | -16.58 | 0.003 |
| hsa-miR-30b-3p | rs841225 | -0.048 | break | C | 154 | -26.33 | 0.274 |
| hsa-miR-30b-3p | rs16963454 | -0.983 | break | C | 150 | -16.75 | 0 |
| hsa-miR-30b-5p | rs6058896 | -0.329 | break | C | 151 | -17 | 0.002 |
| hsa-miR-30b-5p | rs2241648 | -0.591 | decrease | C | 144 | -9.34 | 0.002 |
|  |  |  |  | G | 141 | -10.34 | 0 |
| hsa-miR-30b-5p | rs2241648 | -0.591 | enhance | C | 141 | -10.34 | 0 |
|  |  |  |  | G | 144 | -9.34 | 0.002 |
| hsa-miR-30b-5p | rs1128474 | -0.592 | decrease | A | 157 | -16.51 | 0.076 |
|  |  |  |  | G | 153 | -16.2 | 0.076 |
| hsa-miR-30b-3p | rs10305751 | - | break | C | 140 | -18.64 | 0.555 |
| hsa-miR-30b-5p | rs1042712 | -0.43 | create | G | 145 | -7.46 | 0.002 |
| hsa-miR-142-3p | rs12101610 | -0.029 | decrease | A | 156 | -18.46 | 0 |
|  |  |  |  | G | 152 | -18.46 | 0 |
| hsa-miR-142-3p | rs2409764 | - | create | C | 141 | -15.49 | 0.005 |
| hsa-miR-142-3p | rs2576 | - | create | A | 149 | -12.83 | 0.001 |
| hsa-miR-720 | rs4756026 | -0.074 | break | C | 146 | -17.98 | 0.002 |


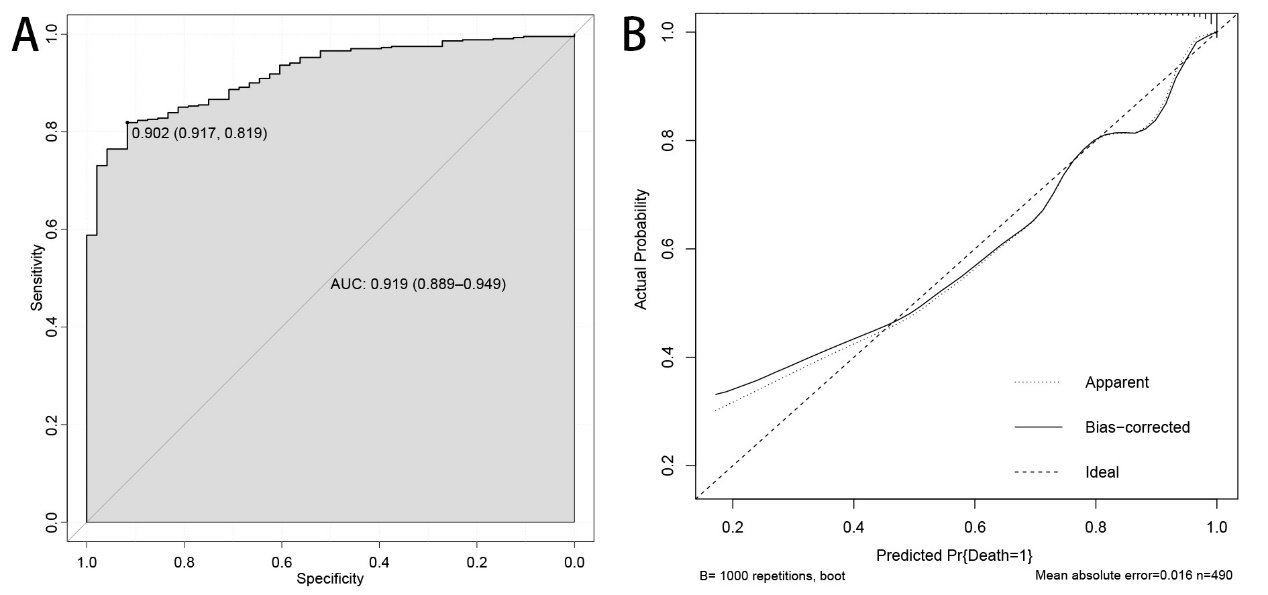


Figure S1: A: The receiver operation characteristic (ROC) curves for the nomogram. B: The calibration curves for the nomogram.
